# The Photobiology of Lutein and Zeaxanthin in the Eye

**DOI:** 10.1155/2015/687173

**Published:** 2015-12-20

**Authors:** Joan E. Roberts, Jessica Dennison

**Affiliations:** Department of Natural Sciences, Fordham University, New York City, NY 10023, USA

## Abstract

Lutein and zeaxanthin are antioxidants found in the human retina and macula. Recent clinical trials have determined that age- and diet-related loss of lutein and zeaxanthin enhances phototoxic damage to the human eye and that supplementation of these carotenoids has a protective effect against photoinduced damage to the lens and the retina. Two of the major mechanisms of protection offered by lutein and zeaxanthin against age-related blue light damage are the quenching of singlet oxygen and other reactive oxygen species and the absorption of blue light. Determining the specific reactive intermediate(s) produced by a particular phototoxic ocular chromophore not only defines the mechanism of toxicity but can also later be used as a tool to prevent damage.

## 1. Introduction

Lutein and zeaxanthin are antioxidants that accumulate in the lens and retina of the human eye [1–4]. These antioxidants protect ocular tissues against singlet oxygen and lipid peroxide damage [5]. Unfortunately, beginning with middle age, antioxidant protection is depleted and this leads to the formation of age-related cataracts and macular degeneration [6].

Increasing the intake of fruits and vegetables high in lutein and zeaxanthin [7–10] has been found to retard age-related cataracts and macular degeneration [11]. In addition, supplementation with lutein and zeaxanthin has been very effective at restoring these important ocular antioxidants [12, 13]. The level and distribution of these carotenoids can be directly and noninvasively measured in the human eye [14–16]. Increasing these carotenoids has been found not only to lower the risk for irreversible blindness [12, 17–20] but also to potentially improve cognitive function in the elderly [21–23].

Determining the specific reactive intermediate(s) produced by a particular phototoxic ocular chromophore not only defines the mechanism of toxicity but can also later be used as a tool to prevent damage. For instance, lutein and zeaxanthin prevent singlet oxygen damage [5], whereas N-acetyl cysteine has been shown to be particularly effective in quenching UV phototoxic damage and inflammation [24, 25]. In this review, we describe the underlying photobiological mechanisms involved in the induction of light-induced damage to the eye and the appropriate and inappropriate antioxidants to protect against such damage.

## 2. Ambient Radiation Ocular Damage

The primary factors that determine whether ambient radiation will injure the human eye are the wavelengths emitted from sunlight or a specific lamp [26] and received by ocular tissues; the intensity of the light; and the age of the recipient.

### 2.1. Wavelength Emitted from Source

Radiation from the sun emits varying amounts of UV-C (220–280 nm), UV-B (280–320 nm), UV-A (320–400 nm), and visible light (400–700 nm) [27]. Most of the UV-C and some short wavelengths of UV-B are filtered by the ozone layer [28]. Artificial light sources emit differing wavelengths of light depending on their spectral distribution [29]. UV radiation contains wavelengths shorter than visible light; the shorter the wavelength, the greater the energy and the greater the potential for biological damage. However, although the longer wavelengths are less energetic, they penetrate the eye more deeply [30].

### 2.2. Wavelength Transmission of Light through the Human Eye

**Figure 1:**
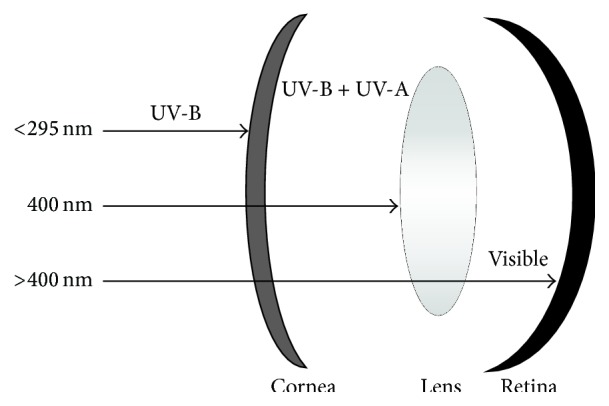
Wavelength transmission of the adult human eye.

In order for a photochemical reaction to occur in the eye, the light must be absorbed in a particular ocular tissue. The primate/human eye has unique filtering characteristics that determine in which area of the eye each wavelength of light will be absorbed [30]. All UV radiation of wavelengths shorter than 295 nm is filtered by the human cornea. This means that the shortest, most energetic wavelengths of light (all UV-C and some UV-B) are filtered out before they reach the human lens. Most UV light is absorbed by the adult lens, but the exact wavelength absorbed depends upon age [31] as shown in Figure 1. The very young human lens transmits UV radiation to the retina, while the elderly lens filters out much of the short blue visible light (400–500 nm) [32] before it reaches the retina. In adults, the lens absorbs UV-B and all the UV-A (295–400 nm); therefore only visible light (>400 nm) reaches the retina. Transmission also differs with species; the lenses of mammals other than primates transmit ultraviolet light longer than 295 nm to the retina [33]. Aphakia (removal of the lens) and implanted Intraocular Lenses (IOLs) after cataract surgery will also change the wavelength characteristics of light reaching the retina [34–37].

### 2.3. Intensity and Mechanism

Ocular damage from light can occur through either an inflammatory response or a photooxidation reaction. Acute exposure to intense radiation, for example, exposure to sunlight reflected from snow (snow blindness), or from staring at the sun during an eclipse [37] or directly staring at an artificial light source that emits UV-A or UV-B [38, 39] causes a burn in the eye similar to sunburn. This induces an inflammatory response in the eye. The initial insult to the tissue provokes a cascade of events that eventually results in wider damage to the cornea, lens, and/or retina [24, 40, 41].

**Figure 2:**
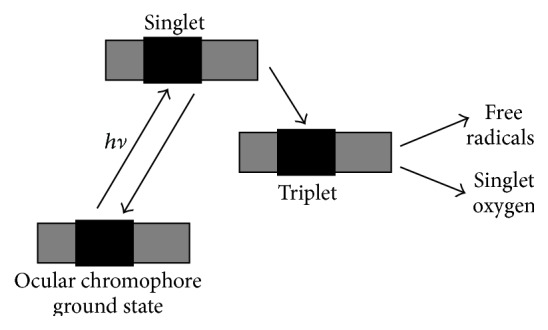
Photooxidation.

Chronic exposure to less intense radiation damages the eye through a photooxidation reaction. In photooxidation reactions, a chromophore in the eye absorbs light and produces reactive oxygen species such as singlet oxygen and superoxide that damage ocular tissues as shown in Figure 2. The chromophore may be endogenous (natural) or exogenous (drug, herbal medication, or nanoparticle that has accumulated in the eye) [27]. If an ocular pigment is excited by ambient radiation to the excited state (singlet) but very quickly (in picoseconds) goes back to the ground state, it will safely dissipate the energy received [42].

## 3. Age and Endogenous Singlet Oxygen Chromophores

As the eye ages, chromophores which were once protective of the eye are modified and become phototoxic. The potential to produce singlet oxygen is measured as a quantum yield. Quantum yield measures the amount of an excited state produced by an amount of light energy used. The higher the number is, the more efficient the chromophore is at making a specific reactive oxygen species. For instance, a chromophore with a Quantum Yield for Singlet Oxygen of 0.10 is a very strong oxidant, while a chromophore with a Quantum Yield for Singlet Oxygen of 0.002 produces negligible amounts of singlet oxygen.

### 3.1. Lens

The primary function of the human lens is to focus light undistorted onto the retina. Although the transmission properties of most of the components of the eye are stable, the transmission properties of the lens change throughout life. The lens is clear for the first 3 years of life and then gradually develops yellow chromophores (3-hydroxy kynurenine and its glucoside). These are endogenous protective agents which absorb UV radiation and safely dissipate its energy [42].

As long as these chromophores are present, neither UV-A nor UV-B radiation reaches the retina, and in this way, the adult human retina is protected against normal levels of UV radiation [43]. However, children are at particular risk for UV damage to the retina because UV is directly transmitted to their retinas [33].

After middle age the protective chromophores 3-hydroxykynurenine and its glucoside are enzymatically converted into the phototoxic chromophores xanthurenic acid and xanthurenic glucoside [44, 45]. These xanthurenic derivatives absorb UV radiation, form triplet states, and produce singlet oxygen [46, 47] with a quantum yield of 0.170. These endogenous singlet oxygen photosensitizers cross-link lens protein [44] and induce apoptosis in lens epithelial cells [45]. There is also an increase in N-formylkynurenine [48, 49] in the lens; it is also an endogenous singlet oxygen photosensitizer. These quantum yields are seen in Table 1.

All of these phototoxic tryptophan derivatives are responsible for UV-A-induced damage to certain target genes [50]. With aging there is also a decrease in the production of antioxidants and antioxidant enzymes in the lens, which would normally quench these reactive oxygen species and prevent damage to the lens. As a result of the increase in phototoxic chromophores concomitant with the loss of antioxidant protection, both the lens epithelial cells and lens proteins are injured, which results in the eventual clouding of the lens, commonly known as a cataract [44].

Phototoxic reactions, whether they are caused by endogenous or exogenous singlet oxygen photosensitizers, can cause a modification of certain amino acids (histidine, tryptophan, and cysteine) [51] and/or a covalent attachment of a sensitizer to cytosol lens proteins. In either case, the physical properties of the protein are changed, leading to aggregation and finally opacification (cataractogenesis). The covalently bound chromophore may now act as an endogenous sensitizer of singlet oxygen, producing prolonged sensitivity to light. Since there is little turnover of lens proteins this damage is cumulative. Any modification in the clarity of the lens impairs both vision and circadian function [52] and has a dramatic effect on retinal function.

### 3.2. Retina

The young retina is at particular risk for damage from UV exposure because the young lens has not as yet synthesized the yellow chromophores that prevent UV transmission to the retina [42, 43]; UV damage to the eye is cumulative and may increase the possibility of developing eye disorders (macular degeneration) later in life [26].

In addition to UV damage, short-wavelength blue visible light (430 nm) damages the retinas of those over 50 years of age through a photooxidation reaction with an accumulated chromophore, lipofuscin [30, 53–56].

Lipofuscin is a heterogeneous material composed of a mixture of lipids, proteins, and various fluorescent compounds. It is mainly derived from the chemically modified residues of incompletely digested photoreceptor outer segments [57]. Photoreceptor cells (rods and cones) shed their outer segments (disc shedding) daily to be finally phagocytosed (digested) by RPE cells. This RPE phagocytosis [58, 59] releases lipofuscin. With age, the rates of lipofuscin formation and disposal become unbalanced [60, 61], resulting in lipofuscin accumulation in the RPE [62, 63].

In response to short blue visible light (430 nm), lipofuscin efficiently produces singlet oxygen and lipid peroxy radicals; there is also some production of superoxide and hydroxyl radicals [64–67]. Lipofuscin is autofluorescent, and in previous studies [68] it was hypothesized that the main phototoxic component of lipofuscin was A2E [*N-retinylidene-N-retinylethanolamine*]. This is a pyridinium bisretinoid produced by the condensation of phosphatidylethanolamine with two moles of all-*trans*-RAL [*trans*-retinal]. However, current studies have proven that, rather than being a photooxidative agent, A2E forms the basis of a natural protective mechanism that removes the strong singlet oxygen photosensitizer all-*trans*- RAL [69] and keeps it from damaging the RPE cells by forming the very weak singlet oxygen inducer A2E [27, 30, 56, 70, 71]. While the quantum yield for lipofuscin [Φ = 0.09] is relatively high, the quantum efficiency for the generation of singlet oxygen by A2E is very low (Φ = 0.0003) [67, 72].  Table 2 gives the quantum yields of these retinal chromophores.

Further* in vivo * mouse studies [55] and human studies using matrix-assisted laser desorption ionization imaging mass spectrometry (MALDI IMS) and FT-ICR tandem mass spectrometry confirm that although A2E accumulation in the retina may be hazardous, the damage done is not through a photooxidative mechanism [73–75]. Another mechanism for A2E toxicity to the retina may be the inhibition of phagolysosomal degradation of photoreceptor phospholipids [76], which would increase the production of lipofuscin [60, 77], a blue light singlet oxygen photosensitizer [66, 67], leading to damage to RPE cells. Because the rods and cones survival is dependent on healthy RPE, these primary vision cells will eventually die, resulting in a loss of (central) vision (macular degeneration) and other retinopathies. Another potential toxic mechanism of A2E that does not involve light is the activation of microglial phagocytosis of photoreceptor cells [78, 79].

## 4. Prevention of Damage by Lutein and Zeaxanthin

Lutein and zeaxanthin are ocular antioxidants of dietary origin [80]. These carotenoids are found in the human lens, [81], retinal pigment epithelium/choroid (RPE/choroid), the macula, the iris, and the ciliary body [2]. Recent clinical trials have determined that age- and diet-related loss of lutein and zeaxanthin enhances phototoxic damage to the human eye, while supplementation of these carotenoids has a protective effect against photoinduced damage to the lens and the retina. The use of improper carotenoids as an antioxidant (*β*-carotene) for quenching light damage to the eye as was used in the AREDS 1 clinical trial is not only ineffective because it does not pass blood ocular barriers but may be hazardous to human health [82, 83].

### 4.1. Structure of Carotenoids in relation to Their Function and Location in the Eye

**Figure 3:**
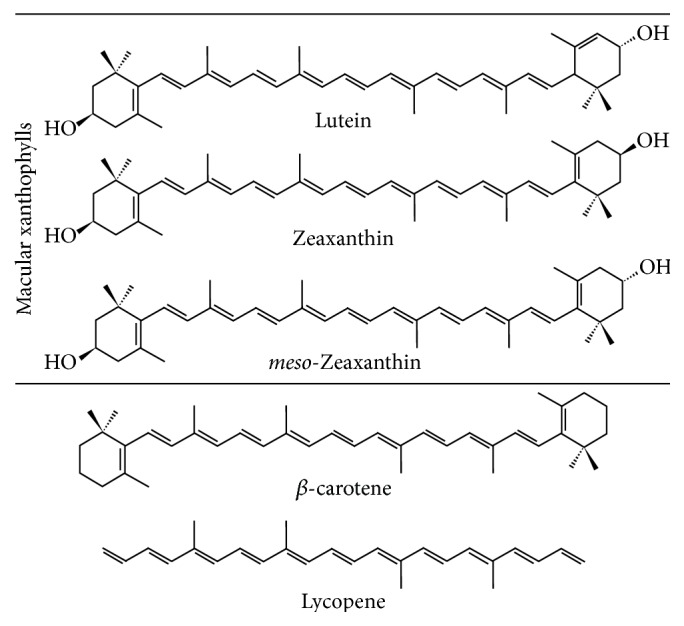
Structures of lutein, zeaxanthin, B-carotene, and lycopene.

Lutein and zeaxanthin have a 40-carbon basal structure, which include a system of conjugated double bonds (alternating double and single bonds) as shown in Figure 3. Chemical structures with extensive conjugated bonds absorb light in the visible range; lutein and zeaxanthin absorb blue visible light (400–500 nm).

Carotenoids that are substituted with hydroxyl (-OH) functional groups are known as xanthophylls. Lutein and zeaxanthin are xanthophylls, and their hydroxyl functional groups permit both lutein and zeaxanthin and their structural isomers to cross both blood-ocular and blood-brain barriers. Other carotenoids (*β*-carotene and lycopene) contain only carbon and hydrogen atoms and do not cross the blood-brain or ocular barriers [84].

### 4.2. Photochemical Mechanism of Protection

**Figure 4:**
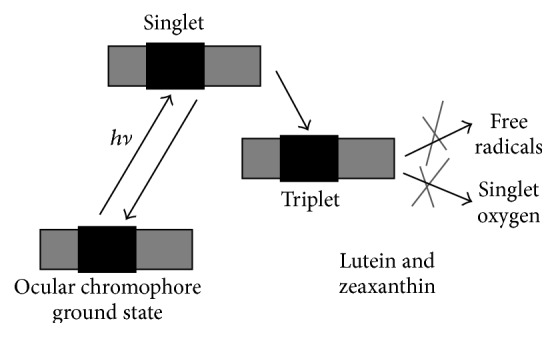
Photochemical mechanism of protection.

Ocular exposure to sunlight, UV, and short blue light-emitting lamps directed at the human eye can lead to the induction of cataracts and retinal degeneration. This process is particularly hazardous after the age of 40 because there is a decrease in naturally protective antioxidant systems and an increase in UV and visible light-absorbing endogenous phototoxic chromophores that efficiently produce singlet oxygen and other reactive oxygen species. The primary mechanism of damage is through a photooxidation reaction. In photooxidation reactions, phototoxic chromophores in the eye absorb light, are excited to a singlet and then a triplet state, and from the triplet produce free radicals and reactive oxygen species which in turn damage the ocular tissues [83, 85]. The phototoxic reactions damage can be prevented by the appropriate antioxidant quenchers as shown in Figure 4.

Lutein and zeaxanthin are naturally accumulating ocular antioxidants that efficiently quench both singlet oxygen and lipid peroxy radicals [86]. Zeaxanthin, with 11 conjugated double bonds, has a higher ability to quench singlet oxygen than lutein (10 conjugated double bonds) as shown in Figure 3 [87].

**Figure 5:**
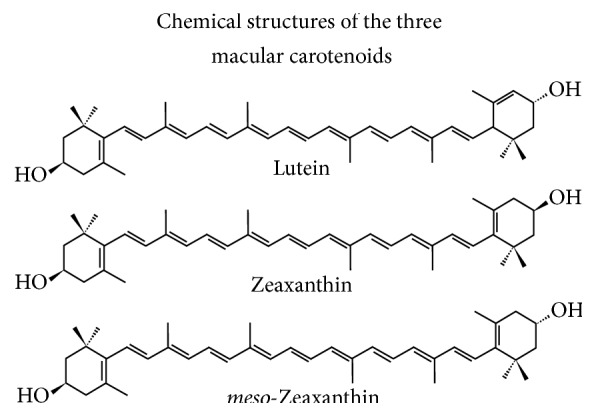
The structures of xanthophyll isomers.

The synergistic action of several ocular antioxidants not only mimics the natural antioxidant protection of the eye (xanthophylls, vitamin E, vitamin C, and glutathione) but also has been found to be most effective. The highly successful synergistic action of zeaxanthin and vitamin E or vitamin C indicates the importance of the antioxidant interaction in efficient protection of cell membranes against oxidative damage induced by photosensitized reactions [88]. Increased levels of both lutein and zeaxanthin were found to reduce age-related nuclear cataracts [89, 90]. Clinical trials with a combination of lutein, zeaxanthin, and its isomer* meso*-zeaxanthin were found to be more protective of the retina than lutein or zeaxanthin alone [12, 91]. This is not surprising as the order of efficiency of quenching singlet oxygen is lutein < zeaxanthin <* meso*-zeaxanthin < all three combined [86, 92]. The structures of these xanthophylls are shown in Figure 5.

### 4.3. Photochemical Mechanism of Prooxidation and Damage by Antioxidants

Both lutein and zeaxanthin are very effective quenchers of singlet molecular oxygen (^1^O_2_) and lipid peroxy radicals. However, in the process, these carotenoids are oxidized to their corresponding radical cations. These cations must be reduced to regenerate the original carotenoid, allowing their reuse as an antioxidant. Vitamin E (*α*- tocopherol) is an antioxidant that can reduce oxidized carotenoids, but in turn, this leaves the tocopherol oxidized [93]. However, the oxidized vitamin E can be reduced and regenerated by vitamin C (ascorbic acid). Vitamin C can then be further reduced by copper and zinc [94, 95]. Without this appropriate combination of oxidizing and reducing agents, antioxidants become prooxidants and can potentially damage the retina and other organs as was found in the AREDS 1 clinical trial [82, 96].

*Summary*. It is essential to determine the specific reactive intermediate(s) produced by a particular endogenous or exogenous photosensitizing agent in each compartment of the eye. This information not only defines the mechanism of toxicity but can also later be used as a tool to prevent damage. For instance, singlet oxygen that forms with the photooxidation of lipofuscin in the aged retina may be quenched by dietary or supplemental lutein and zeaxanthin, thereby preventing damage to the human retina. Using the proper sunglasses to block wavelengths that excite endogenous and exogenous ocular photosensitizers has been shown to limit the singlet oxygen damage to the eye. In the future, gene therapy for retinal dystrophies will be initiated. Ocular imaging techniques using confocal imaging or with adaptive optics are now available. These techniques will allow for direct verification of the physical and metabolic state of the human eye and accurate and digitalized monitoring of any therapeutic benefit of all new treatments against blindness including antioxidant supplements such as lutein and zeaxanthin.

## Figures and Tables

**Table 1:** Quantum yields for singlet oxygen for lenticular chromophores.

	Xanthurenic	NFK
Singlet oxygen	0.17	0.17

	3-OH Kyn	Kynurenine

Singlet oxygen	None	0.006

**Table 2:** Quantum yields for singlet oxygen for retinal chromophores.

	Lipofuscin	*trans*-Retinal	A2E
Singlet oxygen	0.09	0.24	.004
